# Anti-Hepatocellular Carcinoma Effect and Molecular Mechanism of the Estrogen Signaling Pathway

**DOI:** 10.3389/fonc.2021.763539

**Published:** 2022-01-12

**Authors:** Yusheng Guo, Guohui Wu, Junrong Yi, Qin Yang, Wengong Jiang, Shaoqiang Lin, Xiaorong Yang, Xiangsheng Cai, Liufeng Mao

**Affiliations:** ^1^ Scientific Research Center, The First Affiliated Hospital of Guangdong Pharmaceutical University, Guangzhou, China; ^2^ Nephrology Department, The First Affiliated Hospital of Guangdong Pharmaceutical University, Guangzhou, China; ^3^ Clinical Laboratory, The First Affiliated Hospital of Guangdong Pharmaceutical University, Guangzhou, China; ^4^ Center for Medical Experiments, University of Chinese Academy of Science-Shenzhen Hospital, Shenzhen, China

**Keywords:** hepatocellular carcinoma (HCC), estrogen signaling pathway, tumor therapy, molecular mechanism, estrogen receptor

## Abstract

There are significant gender differences in the incidence and mortality of hepatocellular carcinoma (HCC). Compared with men, the incidence and mortality of HCC in women are relatively low. The estrogen signaling pathway, composed of estrogen and estrogen receptors, has been postulated to have a protective effect on the occurrence and development of HCC. There have been multiple studies that have supported anti-HCC effects of the estrogen signaling pathways, including direct and indirect pathways such as genomic pathways, rapid transduction pathways, non-coding RNA, tumor microenvironment, estrogen metabolites, and inhibition of hepatitis infection and replication. Based on the evidence of an anti-HCC effect of the estrogen signaling pathway, a number of strategies have been investigated to determine the potential therapeutic effect. These have included estrogen replacement therapy, targeting the estrogen receptor, key molecules, inflammatory mediators, and regulatory pathways of the estrogen signaling pathway. In this review, we have systematically summarized the latest developments in the complex functions and molecular mechanisms of the estrogen signaling pathway in liver cancer. Furthermore, we have highlighted the potential targets of treatment strategies based on the estrogen signaling pathway in the treatment of liver cancer and the principal obstacles currently encountered for future investigation.

## 1 Introduction

Hepatocellular carcinoma (HCC) is a highly lethal malignant tumor with a growing worldwide incidence that remains a leading cause of cancer-related death. Chronic infection with hepatitis B virus (HBV) and hepatitis C virus (HCV), and nonalcoholic fatty liver disease (NAFLD) are strongly linked to HCC. Traditional treatment methods for HCC offer limited cure options, including surgical resection, local ablative treatments, chemotherapy, targeted therapy and immunotherapy (1). Targeted therapy prevents the growth, progression and metastasis of cancer by interfering with specific molecules. The only systemic agent with proven clinical efficacy for patients with unresectable HCC over the past decade is sorafenib. However, sorafenib has the detrimental characteristic of high-level drug-resistance development, and is therefore associated with poor patient tolerance (2). The estrogen signaling pathway is a signal transduction pathway composed of estrogen and its related receptors. It has multiple functions such as regulation of reproduction, growth and development, and immunity (3). Epidemiological studies have confirmed that females have a lower incidence and reduced mortality from HCC than males, which suggests that the estrogen signaling pathway may play a protective role in the pathogenesis of HCC (4).

The protective effect of the estrogen signaling pathway in the development of liver cancer has been studied and established in pre-clinical models including *in vitro* cellular experiments and animal studies. Regulatory actions of estrogen and the estrogen receptor can antagonize liver cancer through modulation of proliferation, cell cycle, apoptosis and invasive potential of liver cancer cells. In animal models of both castrated and uncastrated males, chemically induced liver cancer can be effectively inhibited with estrogen treatment (5). Our most recent study demonstrated that estrogen could regulate the ERα-36/AKT/Foxo3a signal axis to reduce the transcription of related oxidative stress scavenger enzymes and trigger oxidative stress, thereby inducing liver cancer cells to undergo apoptosis (6). This was the first report of the application of estrogen to HCC cells to induce oxidative stress, an important initial step to further clarify the anti-HCC effect and mechanism of the estrogen signaling pathway.

Dating back twenty years, based on the estrogen signaling pathway, estrogen antagonism was used in both prospective and retrospective studies for treatment of liver cancer, but the results were unsatisfactory (7–10). However, in recent years, estrogen replacement therapy has been applied to the treatment of liver cancer with good results from multiple retrospective studies. These data support an anti-HCC effect of estrogen replacement therapy (11, 12). These new treatment methods based on the estrogen signaling pathway represent a potentially valuable therapeutic strategy in the treatment of liver cancer. However, it is vital to elucidate the functions and regulatory mechanisms of the estrogen signaling pathway as they pertain to hepatocellular cancer.

This review is intended to describe the functions and pathways of the estrogen signaling pathway constituted by estrogen and estrogen receptors, as well as the complex functions and molecular mechanisms of the estrogen signaling pathway in the occurrence and development of liver cancer.

## 2 Overview of the Estrogen Signaling Pathway

Estrogens are important steroid hormones that are responsible for the control of functions of the female reproductive system, and the development of secondary sexual characteristics. They exert their actions by binding to the estrogen receptors (ERs), activating transcriptional processes and signaling events that control gene expression. In addition to regulation of reproductive physiology and function, they influence cell growth, proliferation, immune response and metabolism. The estrogen signaling pathway is mainly composed of estrogens, estrogen receptors, estrogen related receptor (ERR), and a series of target molecules regulated by them.

### 2.1 Estrogen

**Figure 1:**
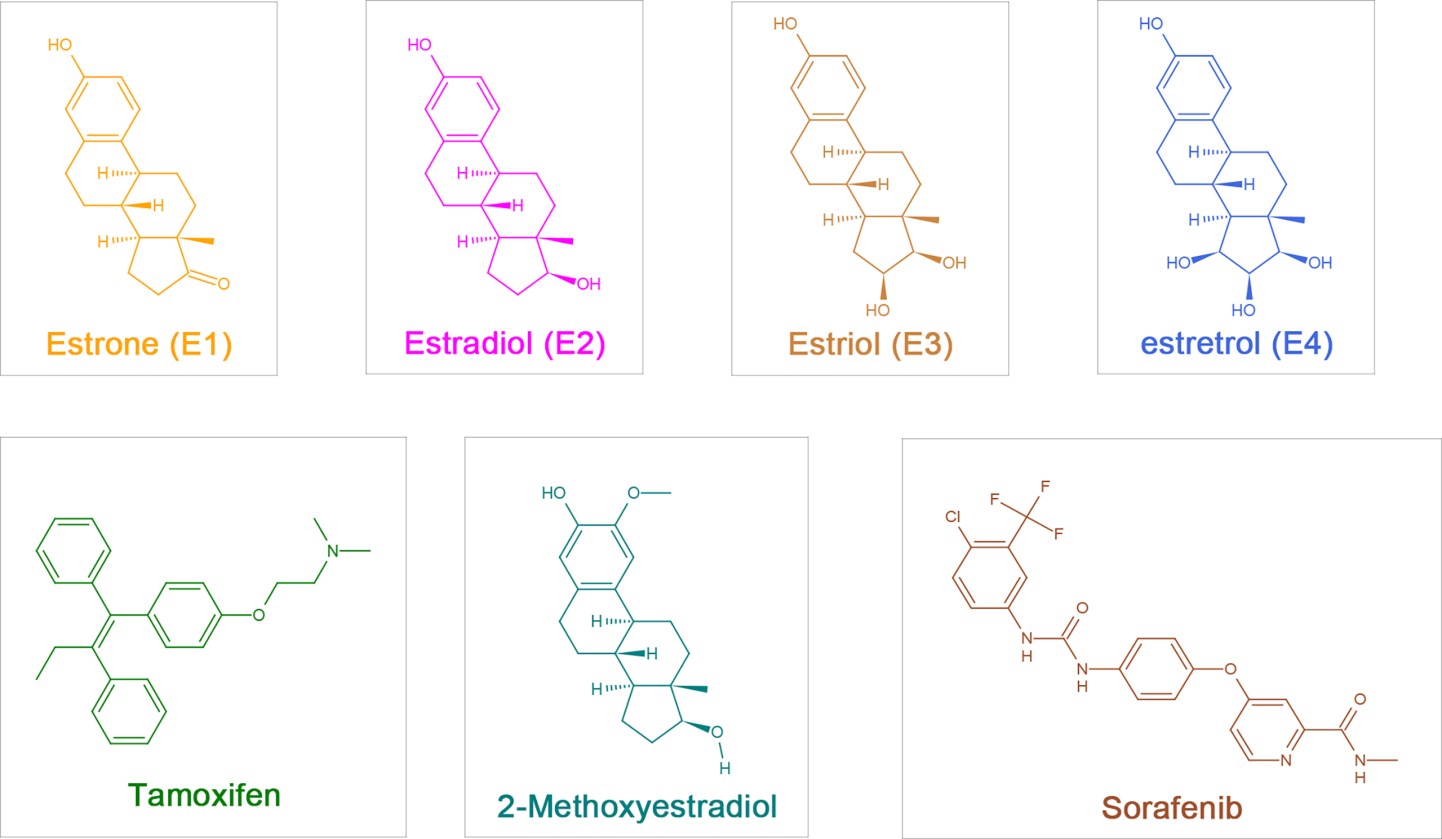
The chemical structure of estrogen, estrogen metabolites and sorafenib. Estrogen:estrone (E1), 17-β-estradiol (Estradiol,E2), Estriol (E3), Estretrol (E4) and Tamoxifen; Estrogen metabolites: 2-Methoxyestradiol (2-ME); Sorafenib.

Estrogens include endogenous estrogens, phytoestrogens, xenoestrogens and selective estrogen receptor modulators (SERMs). Endogenous estrogens are the physiological estrogens that are naturally produced and metabolized in the human body, principally including estrone (E1), 17-β-estradiol (E2), estriol (E3), and estretrol (E4) (**Figure 1**); E2 is the main estrogen hormone in humans due to its predominance and physiological relevance during reproductive years (3).

Selective estrogen receptor modulators (SERMs) are compounds that have tissue-specific activation or antagonism of estrogen receptors. SERMs were originally developed to be used in the treatment of estrogen-dependent tumors such as breast cancer. Among them, tamoxifen has been widely used in the treatment of breast cancer and has been highly successful in the adjuvant setting and in breast cancer prevention (13).

### 2.2 Biosynthesis, Secretion, and Metabolism of Estrogen

In premenopausal women, estrogen is mainly synthesized and secreted by the ovaries, and the granulosa cells in the ovaries synthesize estrogen from androgens. Additionally, there are other organs and tissues that can synthesize estrogen and use it in a paracrine or endocrine manner, such as liver, pancreas, adrenal glands, and adipose and breast tissues. Placental tissue also produces estrogen (E3) during pregnancy (14). In postmenopausal women, circulating estrogen is mainly synthesized and secreted by the adrenal gland and adipose tissue. Aromatase mediates the one-way reaction of the conversion of androgen into estrogen in local tissues, and it is the only enzyme responsible for local estrogen formation, which is essential for maintaining normal estrogen levels in premenopausal and postmenopausal women (15). Estrogen metabolism is principally carried out in liver tissues that highly express members of the cytochrome P450 enzyme superfamily (CYP1A1, CYP1B1, and CYP1A2) that catalyze estrogen hydroxylation. After a series of binding reactions, estrogen becomes water-soluble and is excreted through the urine, feces, and bile. Hydroxylation of estrogen produces catechol estrogens (2-hydroxyestrone, 4-hydroxyestrone, 2-hydroxyestradiol, 4-hydroxyestradiol, and 16α-hydroxyestrone), which have the pharmacological properties of catecholamines and estrogen (16–18).

CYP1B1, which is highly expressed in breast, uterus and ovarian tissues, specifically catalyzes the 4-hydroxylation of estradiol. Because the reduction-oxidation cycle of 4-hydroxyestradiol itself generates free radicals that cause cell damage, the local specific formation of 4-hydroxyestradiol carries great significance for cancerization of breast, uterine and ovarian tissues (17). Catechol-estrogen-3, 4-quinone, the oxidative product of 4-hydroxyestradiol, reacts with specific purine bases in DNA to form depurinating estrogen-DNA adducts. The resulting apurinic sites can lead to oncogenic mutations. Because of this unique carcinogenic reaction, catechol estrogen-3, 4-quinone plays a central role in breast cancer initiation (19).

In addition, catechol-O-methyltransferase can catalyze the conversion of catechol estrogens into methoxy estrogens, which have anti-proliferative properties and the ability to control estrogen synthesis (20). Estrogen metabolites may have either anti-tumor or tumor-promoting effects dependent on their different configurations. These in turn depend on the type of CYP isoforms expressed by target tissues and are tissue specific. Of note, CYP enzymes are regulated by the estrogen signaling pathway, which has physiological significance for maintaining homeostasis of estrogen in local organs (21).

### 2.3 Estrogen Receptor

There are two classical isoforms of estrogen receptor, ERα and ERβ, which are encoded by ESR1 and ESR2 genes, respectively. ERα and ERβ are expressed in a variety of tissues, such as uterus, ovary, breast, liver and brain. The specific isoform and expression level of the ER are the main factors that determine its tissue-specific estrogen responsiveness (22).

**Figure 2:**
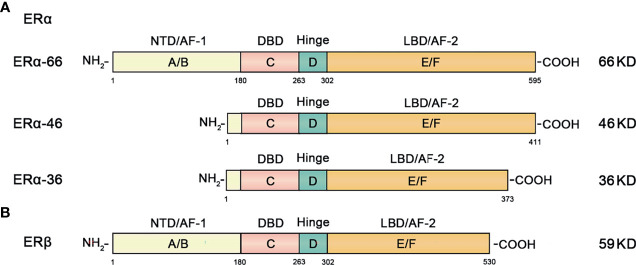
Structural and functional domains of the major classical subtypes of estrogen receptors. **(A)** The three main isotypes of ERα include ERα-66, ERα-46 and ERα-36. The full-length form of ERα-66 has six different structural domains (functional domains): A/B domain (NTD, AF-1), DNA binding domain or C domain (DBD), D domain (hinge region), and ligand-containing binding domain or E/F domain (LBD, AF-2). **(B)** ERβ has 6 different domains (functional domains) similar to ERα.

ERα includes ERα-66, ERα-46, ERα-36 and other isoforms. The classic isoform of ERα is 66KDa ERα-66, which can be divided into 6 different regions in structure (A-F) (**Figure 2**) (23).

ERα-46 is a truncated amino-terminal isoform that lacks the A/B region which encodes transcription activation domain (AF-1). High expression of ERα-46 found in breast cancer reduces the sensitivity of tamoxifen to breast cancer cells (24).

ERα-36 is a recently discovered ERα isoform. It lacks the AF-1 and AF-2 transcriptional activation function domains, retaining only the DNA binding domain (DBD), ligand binding domain (LBD) and hinge region. The overall characteristics of ERα-36 and ERα-46 are similar, but their ends contain a characteristic 27 amino acid domain that is different from the 138 amino acids encoded by ERα-66 and ERα-46 isoform genes (25, 26).

ERβ is an ER subunit that also consists of 6 regions and contains the A-F domain (**Figure 2**). The main difference from ERα is that the amino terminal domain of ERβ is relatively short.

In addition to the classic nuclear receptors ERα and ERβ, the membrane receptor G-Protein-Coupled Estrogen Receptor (GPER) isoform has recently been found on the cell membrane. This is a typical G protein-coupled receptor that acts as a molecular switch signal transducing molecule. Its structure consists of 7 transmembrane α-helix regions, 4 extracellular and 4 cytoplasmic segments. The characteristic structure of GPER allows it to respond quickly to estrogen stimulation (27).

### 2.4 Transduction Pathway of the Estrogen Signaling Pathway

**Figure 3:**
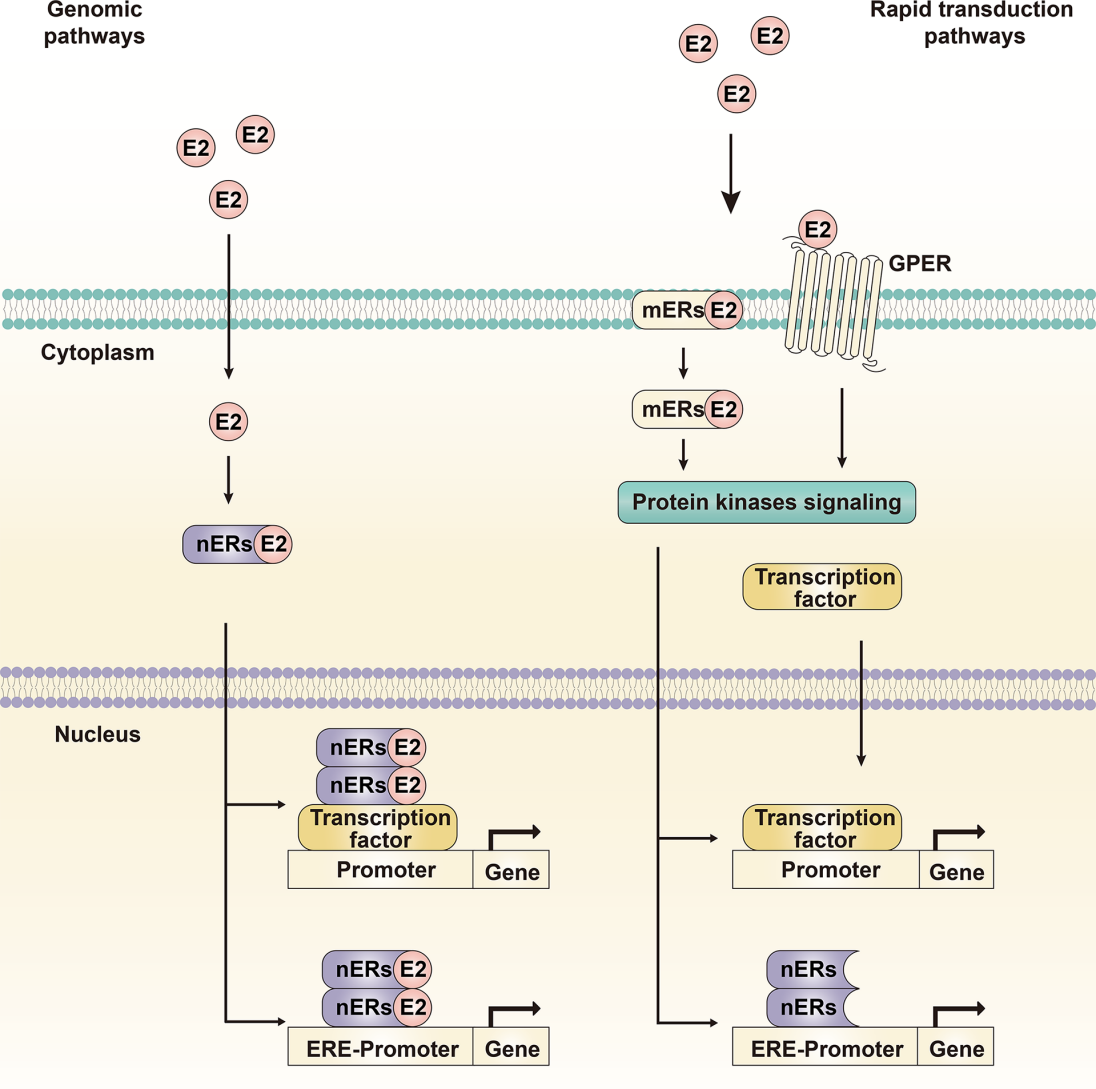
Genomic pathway and rapid transduction pathway. The genomic pathway refers to estrogen binding to nuclear receptors, receptor dimerization and translocation into the nucleus to induce transcriptional changes in estrogen-responsive genes. The rapid transduction pathway refers to estrogen binding to membrane receptors, inducing and activating cytoplasmic events such as intracellular signal transduction cascades and transcription factors. The two pathways crosstalk with each other and mediate the molecular transduction mechanism of the estrogen signaling pathway together.

Estrogen can enter the plasma membrane and interact with the intracellular ER, binding to DNA sequences; this is known as the genomic pathway (**Figure 3**). The main mediating genomic pathway is the classical estrogen nuclear receptors (nERs), including ERα-66, ERα-46, and Erβ (27). Estrogen can bind to nERs to form homodimers and/or heterodimers and translocate to the nucleus, bind DNA on estrogen response elements (EREs) and activate the expression of ERE-dependent genes (28). Under the mediation of stimulating protein (SP-1), the estrogen receptor complex can also interact with other transcription factors and response elements to regulate non-targeted transcriptome activities, and ultimately affect cell proliferation and apoptosis signals (29).

Estrogen can also interact with ERs that do not contain ERE-like sequences but rather activate the intracellular signal transduction cascade, which is called the non-genomic pathway or the rapid transduction pathway (**Figure 3**) (30). The major mediating non-genomic pathways are the new estrogen membrane receptors (mERs), GPER and ERα-36. The estrogen receptors, related pathway proteins, and effector molecules in the two functional pathways crosstalk with each other, leading to differences in the transcriptional activity of specific tissues and physiological processes; these constitute a complex, multidirectional, and multifunctional estrogen signaling pathway.

## 3 Anti-Hepatocellular Carcinoma Effect and Mechanism of the Estrogen Signaling Pathway

As noted previously, global epidemiological studies have demonstrated the consistent prevalence of HCC among men, with worse survival than in females, and have concluded that there is a protective impact of the estrogen signaling pathway against liver cancer. It appears that these anti-tumor effects are mediated by different transduction pathways.

### 3.1 Genomic Pathway

The genomic pathway is the classic transduction pathway of the estrogen signaling pathway, which was also the initial pathway targeted for anti-HCC therapy (**Figure 3**).

#### 3.1.1 Cell-Line Studies

Overexpression of ERα can induce cell transformation of ERα-negative human hepatoma Hep3B cells. ERα can bind to the SP1 protein to form a complex, which then binds to the TNF gene promoter and further induces the expression of active Caspase 3 (an executioner in apoptosis) in a ligand replacement manner (31). The promoter of the MTA1 gene has three and a half ERE binding sites. ERα can inhibit the proliferation and invasion of human HCC cells by down-regulating the transcription of MTA1, and the overexpression of MTA1 weakens the proliferation and invasion of HCC cells and tumor formation *in vivo* by the inhibitory effect of ERα (32). E2 can induce the expression of miR-23a by activating ERα and binding to the regulatory region of miR-23a, or by binding to the regulatory region of p53 to induce the expression of miR-23a. MiR-23a then down-regulates the expression of its target gene XIAP, thereby activating the activity of caspase-3 and inducing apoptosis of liver cancer cells (33). E2 can inhibit the hepatocyte cell cycle marker CDK2 and up-regulate the expression of P53, thereby reducing the viability of hepatocytes and HCC cells (34).

#### 3.1.2 Animal Studies

The anti-liver cancer effect of the estrogen signaling pathway has also been reported in animal experiments. Whereas castration of male mice led to a decrease in the incidence of HCC, castration of female mice led to an increase in the incidence of HCC, lending support to the hypothesis that sex hormones play an important role in the occurrence of liver cancer (35, 36). Exogenous and endogenous estradiol were shown to inhibit the occurrence of chemically induced liver cancer, and ER was reportedly involved in inhibiting the early malignant transformation of liver cancer (5). Protein tyrosine phosphatase receptor type O (PTPRO) is one of the receptor types of phosphotyrosine phosphatase (PTP), known to be a tumor suppressor in various cancers. PTPRO was shown to down-regulate the signal transduction that depends on the dephosphorylation of JAK and PI3K and the transcriptional activity of STAT3, thereby inhibiting the occurrence and development of tumors. ERα was used as a transcription factor of PTPRO to up-regulate its expression and enhance its tumor suppressor effect (37). Compared with male mice, female mice injected with diethylnitrosamine (DEN) showed fewer foci of hyperplasia and slower onset of HCC, smaller tumors, higher differentiation, and fewer metastases. Compared with normal male mice, those injected with DEN after castration reduced the expression of cyclin E kinase and enhanced hepatocyte apoptosis. While estradiol and progesterone enhance these effects, the cyclin E kinase activity of normal female mice is lower than that of male mice. The use of testosterone in ovariectomized female mice up-regulates cyclin E, activates cyclin E kinase, and accelerates the occurrence of liver cancer (34). Some researchers have suggested that Foxa faultidirectional, and multifunctional estrogen signalctor and its target are the core of HCC sexual dimorphism. There are obvious gender differences in HCC mice induced by DEN, and the defects of Foxa1 and Foxa2 can reverse this gender difference relative to the incidence of liver cancer. Further studies have found that Foxa1 and Foxa2 can mediate and enhance the regulation of ERα or androgen receptor (AR) on target genes during the formation of liver cancer, thereby inhibiting (ERα) or promoting (AR) the development of liver cancer (38).

### 3.2 Rapid Transduction Pathway

**Figure 4:**
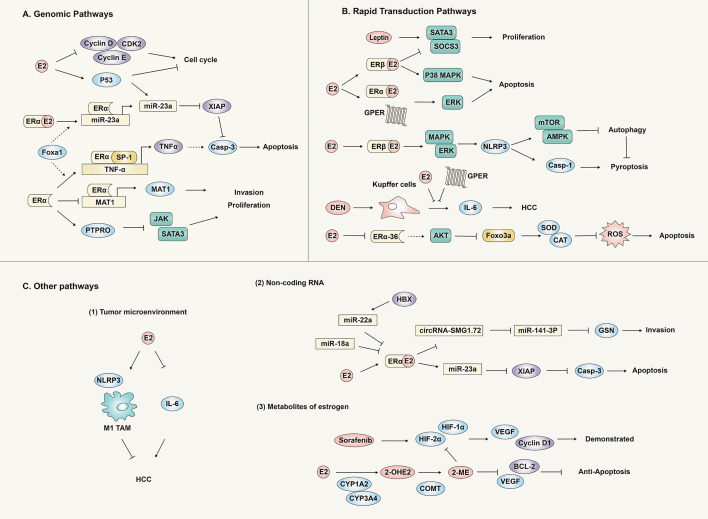
Anti-hepatocellular carcinoma effect and mechanism of the estrogen signaling pathway. Estrogen plays an antagonistic role against liver cancer through genomic pathways **(A)**, rapid transduction pathways **(B)** and other pathways **(C)** (tumor microenvironment, non-coding RNA and metabolites), inhibiting the proliferation, invasion and migration of liver cancer tissues or cells.

In addition to the classic genomic pathway, recent studies have found that the rapid transduction pathway is also an important avenue for estrogen signal transmission to produce an anti-liver cancer effect (**Figure 4**).

Leptin is a hormone secreted by white adipose tissue, that can promote the development of liver cancer. E2 and ER antagonize the carcinogenic effect of leptin in HepG2 cells by inhibiting cell proliferation and stimulating apoptosis. This is due to the reversal of leptin-induced SOCS3/STAT3 changes and the increase of p38/MAPK by activating ER-β. Similarly, activation of ER-α and GPER can also increase ERK expression. These findings provide evidence that various estrogen receptors play different roles and mechanisms in the occurrence of HCC (39).

E2 has been shown to significantly inhibit the malignant behavior of HCC cells through the up-regulation of NLRP3 inflammasomes mediated by the ERβ/MAPK signal pathway (40). Further research found that the NLRP3 inflammasome inhibited the protective autophagy of tumors through the E2/ERβ/AMPK/mTOR pathway. In general, E2 can inhibit the onset of HCC by activating caspase 1-dependent apoptosis and inhibiting protective autophagy (41).

In the liver, IL-6 is an important inducer of acute phase response and infection defense, a mitogen of hepatocytes, mediating liver regeneration and other functions, and is related to the metabolic function of the liver. IL-6 is crucial for maintaining hepatocyte homeostasis (42). However, studies have found that the continuous activation of the IL-6 signaling pathway is harmful to the liver, and that IL-6 can promote the occurrence of liver cancer through multiple steps (43–45). IL-6 mediates liver damage and compensatory hyperplasia caused by the carcinogen DEN, and ultimately leads to tumors. The carcinogen DEN mainly promotes the production of IL-6 by Puffer cells (KCs) through MyD88. E2 can inhibit the secretion of IL-6 in KCs and reduce hepatocyte damage and malignant lesions induced by DEN (46).

The activity of Foxo3a is closely related to the oxidative stress response in cells. Activated Foxo3a reduces oxidative stress by binding to the promoters of genes encoding manganese superoxide dismutase (Mn-SOD) and catalase. Our most recent study demonstrated that E2 could reduce the expression of ERα-36 in liver cancer cells and increase phosphorylation of Akt and Foxo3a, preventing Foxo3a from entering the nucleus, thereby preventing synthesis of Mn-SOD and catalase, triggering oxidative stress and ultimately apoptosis. Oxidative stress (defined as excessive production of reactive oxygen species) is one of the important links in the occurrence and development of many diseases, but recently researchers have harnessed this process to develop anti-tumor oxidative therapy and pro-oxidant treatments. The methodology targets the tumor and causes accumulation of active oxygen within it, killing the tumor cells. To our knowledge, we produced the first report that estrogen induces oxidative stress by regulating Foxo3a, which helped clarify the anti-tumor effect and mechanism of the estrogen signaling pathway through the oxidative stress response (6).

GPER is an estrogen membrane receptor located in the plasma membrane that has low expression in liver cancer tissues. In the mouse liver cancer model induced by the carcinogen DEN, knock out of the GPER gene can significantly promote the occurrence of liver cancer, accompanied by immune cell infiltration, fibrosis, and the production of inflammatory factors (such as IL-6). Furthermore, the selective GPER agonist G-1 can reduce the expression of IL-6 in bone marrow-derived macrophages, but this effect is inhibited by GPER knockdown. However, *in vitro* experiments have shown that the viability and proliferation of liver cancer cells were not directly affected by GPER (47). These results indicated that GPER could inhibit HCC through regulation of the inflammatory response rather than direct action on tumor cells. However, a recent study found that GPER was significantly down-regulated in HCC tissues compared with matched non-tumor tissues. Compared with GPER negative patients, GPER positive HCC patients were significantly associated with female sex, HBsAg negative, small tumor size, low serum AFP levels, and longer overall survival. GPER/EGFR/ERK signaling triggered by GPER specific agonist G1 played a crucial role in decreasing the tumor viability of HCC, both *in vitro* and *in vivo*. Clinical analysis indicated that simultaneous high expression of GPER and phosphorylated ERK (P-ERK) predicted improved prognosis of HCC. These findings suggest that specific activation of the GPER/ERK axis could be a therapeutic target for HCC (48). Therefore, activation of GPER could serve as a potential strategy for prevention and treatment of HCC.

### 3.3 Other Pathways

In addition to the classic genomic pathways and rapid transduction pathways, the estrogen signaling pathway was recently discovered to exert anti-liver cancer effects through multiple pathways such as tumor microenvironment (TEM), non-coding RNA (ncRNA) regulation/small molecule RNA, and metabolites (**Figure 4**).

#### 3.3.1 Tumor Microenvironment

Stromal cells and cytokine-related components of the tumor microenvironment tend to promote tumor cell proliferation, invasion and metastasis, through the action of tumor-associated macrophages (TAM), bone marrow-derived suppressor cells (MDSC), tumor-associated neutrophils (TANs), cancer associated fibroblasts (CAF) and regulatory T cells (Tregs). These immunosuppressive cells can suppress the body’s own immune response thereby reducing the effect of immunotherapy, which has opened a new field of research in the treatment of liver cancer. Typically, immune cell regulation by the estrogen signaling pathway enhances normal immune response. Similarly, recent studies have reported that estrogen can regulate the microenvironment of liver cancer to exert its anti-tumor effect (49, 50).

E2 has also been shown to inhibit tumor growth by regulating the polarization of macrophages. The mechanism involves E2 activation of ER-β that interacts with ATPase coupling factor 6 in the presence of IL-4 to up-regulate SOCS1, thereby inhibiting JAK1-STAT6 signal pathways, which attenuates the selective activation of macrophages and the growth of HCC tumors (51).

NLRP3 inflammasome is an intracellular multi-protein complex that participates in the innate immune response to pathogens and other harmful factors. As noted previously, estrogen can inhibit the protective autophagy of tumors by regulating the NLRP3 inflammasome, ultimately inhibiting the progression of HCC (41).

The estrogen signaling pathway has multiple, complex relationships with the tumor microenvironment. Studies have confirmed that estrogen can inhibit tumor progression by regulating the M1 polarization of macrophages, NK cells, CD8+ T cells, Th1 cells and the resultant inflammatory cytokines, IFNγ, TNFα, IL-12. However, estrogen can also regulate the formation of an immunosuppressive microenvironment that can promote tumor immune evasion by regulating CAF, MDSC, Treg cells, Th2 cells and related cytokines IL-4, IL-6 and other components (50, 51). In breast and ovarian cancer as well as other estrogen-dependent tumors, estrogen has been proven to enhance the immunosuppressive microenvironment thereby promoting tumor development (50). Nevertheless, the complete picture of tumor-specific regulation effects and mechanisms of the estrogen signaling pathways on the tumor microenvironment are still not fully clarified. A more complete understanding of the specific mechanisms of the tumor microenvironment regulated by the different functional pathways of the estrogen signaling pathway will most certainly facilitate the development of new treatment strategies related to the estrogen signaling pathway, particularly enhancing immunotherapy (52).

#### 3.3.2 Non-Coding RNA

With the development of high-throughput RNA sequencing technology and bioinformatics, an ever-enlarging number of ncRNA sequences and their functions have been discovered. Such non-coding protein sequences such as ncRNA perform important biological functions in cells. lncRNA, miRNA and eRNA are all non-coding RNAs, proven to play important functions in the occurrence and development of tumors. Critically important, the estrogen signaling pathway can also regulate the occurrence and development of tumors through ncRNAs (53–55).

miRNA is a short non-coding RNA with a base length of 19-25 bp, which acts as a post-transcriptional regulator of gene expression by mediating the degradation and/or translational inhibition of its target mRNA. A single miRNA can target hundreds of mRNAs and affect the expression of many genes through multiple interaction pathways. Multiple studies have reported the involvement of miRNA in the protective effect of the estrogen signaling pathway on liver cancer (53).

The miR-545/374a cluster is a microRNA cluster encoded by the Ftx gene, found to be highly expressed in HBV-related HCC tissues, and is associated with a poor prognosis of HCC patients (56). This cluster originates from tumor tissues, and its expression is positively regulated by HBV infection and may be induced by HBx expression, suggesting that it could potentially be a new diagnostic screening marker for HCC. As might be anticipated, further research found that the miR-545/374a cluster of male HCC patients was much higher than female HCC patients. The target gene prediction program found that estrogen-related receptor γ (ESRRG) was a potential target gene of miR-545/374a, and ESRRG and expression of miR545 are inversely related (56).

Gelsolin (GSN) is an important molecule that mediates metastasis and invasion of liver cancer cells. ERα can bind to the 5’promoter region of SMG1 to inhibit the transcriptional expression of circRNA-SMG1.72, thereby inhibiting the invasion of HCC cells. CircRNA-SMG1.72 can inhibit the expression of miR-141-3p by producing cavernous bodies, which can in turn target the binding of GSN mRNA to reduce the expression of GSN. The end effect, ERα can inhibit the invasion of HCC cells through the ERα/circRNA-SMG1.72/miR-141-3p/GSN signaling pathway (57).

Using miRNA PCR array technology, E2 treatment resulted in up-regulation in 25 and down-regulation in 10 miRNAs of apoptotic HCC cells. Further studies found that E2 could induce miR-23a expression by activating ERα and binding to the regulatory region of miR-23a, or by binding to the regulatory region of p53 to induce miR-23a expression. The effect was to down-regulate the expression of its target gene XIAP, thereby activating caspase-3, inducing apoptosis of liver cancer cells (33).

In addition to the estrogen signaling pathways that can regulate miRNAs to exert anti-liver cancer effects, other studies demonstrated that some miRNAs could act on estrogen signaling pathways ultimately to inhibit anti-liver cancer effects. miR-18a was found to be highly expressed in female HCC patients. Overexpression of miR-18a reduced the expression of ERα and promoted the proliferation of liver cancer cells (58). The gene ESR1 encoding ERα is one of the targets of miR-18a, and miR-18a can inhibit the translation of ERα by binding to its mRNA in the 3’untranslated region. The initial regulatory factor of miR-18a might represent the abnormal expression or mutation of p53, and accumulation of these factors might reduce tumor protection of the estrogen signaling pathway in the development of female liver cancer (58). miR-221 has been found to be highly expressed in various solid and hematological malignancies. Its involvement in the occurrence and development of tumors has been extensively studied and is expected to become a biomarker and therapeutic target for a variety of tumors (59). In liver cancer cells, HBx can promote the proliferation of liver cancer cells by inhibiting ERα and increasing the expression of miR-221. Moreover, further studies found that miR-221 could inhibit ERα expression by directly binding to ERα, resulting in the proliferation of HCC cancer cells and acting as a tumor promoter (60).

#### 3.3.3 Metabolites of Estrogen

In addition to the anti-liver cancer effects mediated by estrogen and estrogen receptors, estrogen metabolites and derivatives also have special anti-tumor effects in liver cancer.

The liver is the main site of estradiol metabolism. Cytochrome P450 CYP1A2 in the liver can convert estradiol into 2-hydroxyestradiol, and then interaction with the enzyme catechol-O-methyltransferase (COMT) will further methoxylate it to produce 2-methoxyestradiol (2-ME) (61). HCC cells express low or almost no CYP1A2. Overexpression of CYP1A2 can increase the level of the estradiol metabolite 2-ME and enhance the inhibitory effect of estradiol on HCC nuclear xenograft tumors. Both *in vitro* and *in vivo* studies have found that 2-ME could reduce the expression of vascular endothelial growth factor (VEGF) and Bcl-2, promote cell cycle arrest and apoptosis of liver cancer cells; the overall effect: inhibition of liver cancer cell proliferation and tumor growth (61).

Sorafenib is a well-accepted and widely used target drug, approved for the treatment of advanced HCC. Its clinical efficacy, however, has been limited due to drug resistance. The adaptive response of tumor cells to hypoxia is an important mechanism of tumor resistance (62). In liver cancer cells, sorafenib down-regulates the expression of HIF-1α, but up-regulates the expression of HIF-2α, with a final effect of changing the hypoxic response from the HIF-1α-dependent to the HIF-2α-dependent pathway. It promotes the expression of VEGF and Cyclin D1, both being downstream molecules of HIF-2α, and ultimately causes hypoxic HCC cells to lose sensitivity to sorafenib. 2-ME can reduce the expression of HIF-1α, HIF-2α and the downstream molecules VEGF, LDHA and Cyclin D1, to increase the sensitivity of hypoxic HCC cells to 2-ME. *In vivo* and *in vitro* experiments have shown that 2-ME and sorafenib synergistically inhibit the proliferation of HCC cells, induce apoptosis, and inhibit tumor angiogenesis (63).

## 4 The Role of the Estrogen Signaling Pathway in Viral Liver Cancer

Chronic infection with hepatitis B and C viruses is the principal risk factor for liver cancer. With infection, they cause inflammation, oxidative stress, and often delayed fibrotic reactions, eventuating into cirrhosis. This is accompanied by the appearance of local hypoxia, rearrangement of tissue structure (epithelial mesenchymal transition, EMT) and angiogenesis (64). Chronic viral hepatitis can share characteristics of other viral infections, such as affecting the expression of host genes through processes such as genetic changes, DNA repair inhibition, and microRNA differential expression. These changes include the up-regulation of factors involved in “stemness“, indicating that both viruses may promote the development of HCC by promoting stemness (65). In addition to gender differences in the incidence and mortality of liver cancer, significant gender differences have also been found in the prevalence of HBV chronic infection and HBV-related HCC, lending support to the influence of sex hormones in HBV-related HCC (66, 67).

**Figure 5:**
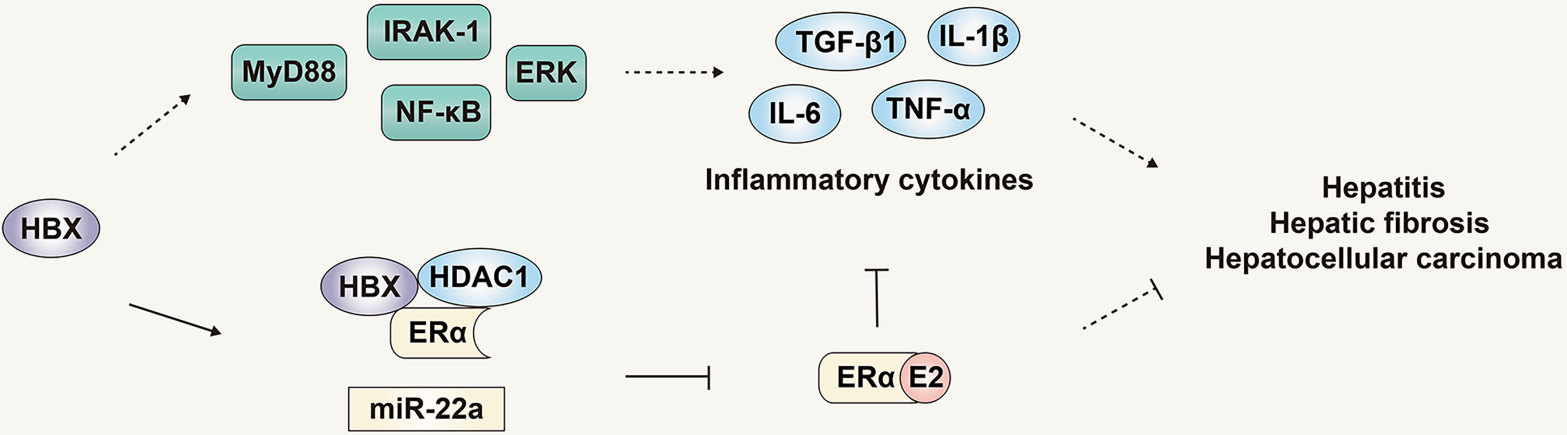
The role and mechanism of the estrogen signaling pathway in viral liver cancer. The estrogen signaling pathway can inhibit the progress of HBV-related HCC by regulating inflammatory mediators. The products of HBV can in turn counteract the estrogen signaling pathway and inhibit its protective effect.

Chronic inflammatory infiltration is an important factor in the progression of HBV infection to HBV-related HCC. It has been confirmed that hepatitis-related inflammatory cytokines such as IL-6, IL-1α and IL-1β are key inflammatory mediators that stimulate the development of HCC (65, 67). In hepatocytes and liver cancer cells, the expression of HBx can activate the downstream signaling proteins IRAK-1, ERKs/p38 and NF-κB of MyD88, thereby promoting the synthesis and secretion of IL-6 and promoting the progress of HBV-related HCC (68). In addition, HBV infection can increase the expression of TGF-β1, IL-1β, TNF-α CTGF and PDGF, thus changing the liver microenvironment and promoting the progression of liver fibrosis (69). Therefore, multiple pathways have been briefly summarized that indicate HBV infection can promote the progress of HBV-related HCC. Counterbalancing these, the estrogen signaling pathway can inhibit the progress of HBV-related HCC by regulating various pathways such as related signal proteins and inflammatory mediators. As noted previously, in addition to inhibiting the secretion of IL-6 by Kupffer cells (46), the estrogen signaling pathway can also regulate the expression of IL-6 by regulating STAT3 and NF-κB signaling molecules. The estrogen signaling pathway can also activate the promoter of ERα binding to IL-1α to reduce its expression (70), thereby regulating the development of HBV-related HCC (**Figure 5**).

Although the estrogen signaling pathway that can regulate progression of HBV infection and HBV-related HCC, the genome and products of HBV can in turn counteract the estrogen signaling pathway and inhibit its protective effect. HBx was reported to inhibit the transactivation function of ERα by forming a trimeric complex with ERα and HDAC1, inhibiting the function of the estrogen signaling pathway, and HDAC inhibitors can reduce the inhibition of HBx on the transactivation function of ERα (71). Moreover, HBx can increase the expression of miR-221, targeting the ERα gene, inhibiting expression of ERα, ultimately inhibiting the protective effect of the estrogen signaling pathway on liver cancer (**Figure 5**) (60).

Increasingly, studies have revealed the protective effect and mechanism of the estrogen signaling pathway in the development of HBV-related HCC. Most importantly, the effect of the estrogen signaling pathway on HBV infection and HBV-related HCC is not a single pathway or single effect, but multiple pathways and multiple effects. However, HBV infection can counteract the estrogen signaling pathway and antagonize its protective effects.

Of overall HCV-infected patients, men and postmenopausal women are more likely to develop liver cirrhosis and liver cancer than premenopausal women. Menopause seems to be associated with acceleration of liver fibrosis in HCV-infected women, whereas estrogen replacement therapy can prevent this progression (72).

Comparing liver tissues of normal people, HCV-related cirrhosis and HCV-related HCC patients, the expressions of ERα and ERβ in liver tissues of patients with HCV-related liver disease were increased to varying degrees, but the expression patterns of ERα and ERβ in the cytoplasm and nucleus were different (73). Similarly, the expression of phosphorylated NF-κB and cyclin D1 were significantly higher in tissues with HCV-related liver disease. In HCV-related HCC, the nuclear ER subtype and nuclear cyclin D1 expression were positively correlated, while the cytoplasmic ER subtype was negatively correlated with cytoplasmic phosphorylated IKK. These results indicated that the dysregulation of ER subtype expression and cell sub-localization following chronic HCV infection could lead to the development of HCV-related cirrhosis and HCV-related HCC.

## 5 Controversy: The Role and Mechanism of the Estrogen Signaling Pathway in Promoting Liver Cancer

In contrast to the multiple studies and different pathways elucidating the protective and preventive roles of the estrogen signaling pathway in liver cancer, some studies have concluded the direct opposite: the estrogen signaling pathway promotes the development of liver cancer. Careful review of these studies reveals the apparent dual role of the estrogen signaling pathway is a function of the different subtypes of the estrogen receptor and the different functional pathways mediated by the spliceosome (74).

ERα-36 is a newly discovered non-classical ERα isoform. Compared with nuclear receptors ERα-66, ERα-36 lacks AF-1 and AF-2 transcription activation domains but retains the DNA binding domain and dimerization domain (**Figure 2**), and ERα-36 is mainly expressed in the cytoplasm and plasma membrane. It mediates non-genomic pathways (or fast signaling pathways) and plays an important role in mitogenic estrogen signaling (75, 76). Of specific relevance to this controversy, the non-genomic transmission pathway mediated by ERα-36 inhibits the genomic transmission pathway mediated by classical nuclear receptors (76).

Initially, the function of ERα-36 was investigated in breast cancer research. ERα (ERα-66) is the most widely used marker for diagnosing human breast cancer, and according to the presence or absence of ERα-66, breast cancer is classified as ER-positive or ER-negative. Anti-estrogens (such as tamoxifen) have been first-line treatments for advanced ER-positive breast cancer. However, recent reports have found that breast cancer is prone to resistance to anti-estrogen therapy, which has an important relationship with ERα-36 (76).

A retrospective study showed that breast cancer patients with high levels of ER-α36 experience less benefit from tamoxifen than those with low levels of ERα-36 expression, and Erα-36 expression is significantly correlated with Her2/Neu expression. These results indicated that ERα-36 might mediate the resistance mechanism of breast cancer to anti-estrogen therapy (77). ERα-36 has been shown to activate downstream signaling pathways through HB-EGF, SRC, EGFR, HER2, IGF-1R, and ultimately to induce the transcription of growth-promoting genes such as c-Myc and Cyclin D1, and finally to stimulate tumor cell proliferation. ERα-36 is also highly expressed in some tumor stem/progenitor cells and participates in their cell maintenance (76). These studies have indicated that this new type of estrogen receptor, ERα-36, mediates an important regulatory role in the occurrence and development of tumors in the estrogen signaling pathway. As such, it serves as a marker for poor prognosis in breast cancer.

The role of ERα-36 in liver cancer has also been explored. In a study of ERα-66 and ERα-36 mRNA expression in non-tumor, cirrhotic and malignant liver tissues and HCC cell lines, it was found that ERα-66 was highly expressed in non-tumor tissues, cirrhotic tissues showed a lower level, whereas its expression decreased or became undetectable in HCC tissues and cell lines. In stark contrast, the expression level of ERα-36 showed just the opposite trend to ERα-66. A high expression of aromatase is an important factor in liver malignancy. It is noteworthy that the expression level of aromatase has the same trend as ERα-36, but opposite to ERα-66. These results emphasize that the principal expression of either ERα-66 to ERα-36 in liver tissues may be directly correlated to the development and/or progression of HCC (78).

**Figure 6:**
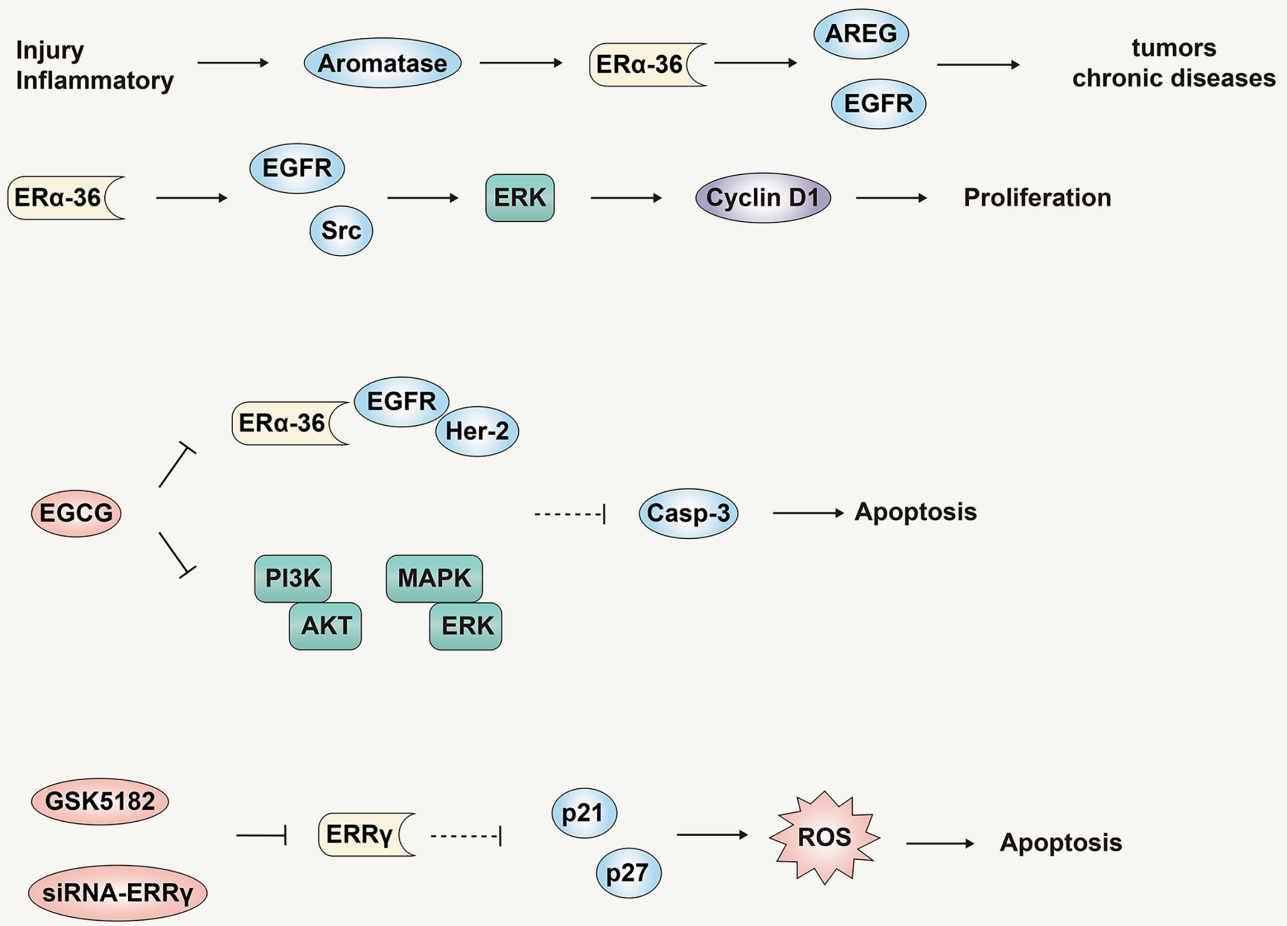
The role and mechanism of the estrogen signaling pathway in promoting liver cancer. ERα -36 is a novel non-classical ERα isotype, which may mediate the estrogen signaling pathway to promote liver cancer. Antagonistic treatment of ERα -36 and ERRγ is expected to become a new therapy for liver cancer.

Researchers have proposed a new model in which tissue damage and/or inflammatory diseases activate the aromatase-estrogen-ARGE-EGFR axis, eventually leading to liver, breast, and prostate cancers, and other chronic diseases such as diabetes (**Figure 6**) (79), obesity, Alzheimer’s disease and heart disease. One such study found that in liver cells, the expression of NF2 was positively correlated with the expression of aromatase, amphiregulin (AREG) and ERα-36, and had a direct correlation with the degree of malignancy, but was inversely correlated with expression of ERα-66. Although estradiol treatment could induce a significant decrease in NF2 expression in HA22T and Huh7 cells, no changes were observed in HepG2 cells. This effect may be negatively related to the expression and activity of aromatase. Aromatase expression in normal and cirrhotic tissue cells is low, while its expression in liver cancer tissues is high. In addition to detecting high levels of ERα-36 in HCC tissues and cells, recent studies have revealed the role and mechanism of ERα-36 in the progression of HCC (80). In HCC cells with high expression of ERα-36, the rapid transduction pathway mediated by ERα-36 can be activated by estrogen, which in turn activates EGFR/Src/ERK signal regulation and up-regulates the expression of CyclinD1. In addition, the ERα-36/EGFR signal axis plays an important role in maintaining and actively regulating the growth of HCC tumor cells (**Figure 6**).

EGCG is a natural product with potential anti-cancer properties and has a dose-dependent inhibitory effect on HCC cells that highly express ERα-36 (81). EGCG can activate expression of p-ERK and Caspase-3 by inhibiting the ERα-36/EGFR/Her-2 feedback loop and the PI3K/Akt and MAPK/ERK pathways. This culminates in the induction of HCC cell apoptosis and proliferation inhibition. As noted previously, our most recent study demonstrated that estrogen can down-regulate ERα-36 expression and activate the AKT/Foxo3a signaling axis, triggering oxidative stress and ultimately apoptosis in HCC cells (**Figure 6**). In summary, multiple studies have identified ERα-36 and its mediated rapid transduction pathway might present a logical target for anti-liver cancer drugs.

In addition to the new receptor ERα-36, estrogen-related receptor γ (ERRγ) may also mediate the cell growth and tumorigenesis of various tumors (82). Compared with normal tissues, the expression level of ERRγ in HCC tissues is higher, and the expression level of ERRγ is also closely related to the pathological tumor grade, metastasis and poor prognosis. In addition, siRNA-ERRγ or GSK5182 (ERRγ antagonists) can induce cell cycle arrest and oxidative stress by increasing the expression of p21 and p27, thereby inhibiting the growth and proliferation of HCC cells (**Figure 6**). These results show that, similar to ER-α36, ERRγ might be a potential biomarker for liver cancer, and antagonistic therapy targeting ERRγ is expected to become a new type of therapy for liver cancer.

## 6 Clinical Application and Results of Estrogen Therapy for Liver Cancer

Mounting study results lend support to the application of anti-liver cancer effects of the estrogen signaling pathway to the development of anti-liver cancer therapies. Although anti-estrogen therapy for liver cancer dates back more than 20 years, the early attempts failed to achieve satisfactory results.

As a selective estrogen receptor modulator (SERM) that competes with estrogen for the ER, tamoxifen was used in a small, prospective controlled trial in 1990 that showed it could be effective in treating unresectable or incurable liver cancer (10). Only 38 patients with inoperable HCC were included, but the survival time of the tamoxifen-treated group was statistically prolonged with a survival rate at 12 months of 22% (control, 5%). However, a subsequent large randomized controlled trial showed that tamoxifen did not prolong liver cancer patient survival (9). As an alternative, higher doses of tamoxifen were proposed intending to inhibit HCC in an ER-independent manner (83). To address this, a double-blind randomized trial from the Asia-Pacific region showed that high-dose tamoxifen did not prolong survival of patients with advanced HCC. In fact, on the contrary, its negative impact could increase as the dose was increased (8). Other studies suggested that tamoxifen might have survival benefit for HCC patients who did not have severe liver insufficiency (7). This seemed to restrict anti-estrogen therapy only for early liver cancer and might not be effective for advanced liver disease. According to large trials and Cochrane systematic reviews, tamoxifen was not deemed beneficial in HCC for overall survival or quality of life (84). Therefore, tamoxifen does not justify further testing in HCC, nor should it be used clinically.

Current research results show that the reason for the failure of tamoxifen may be closely related to the main expression of ERα-66 to ERα-36 in HCC tissues. Compared with ERα-66, ERα-36 lacks two transcriptional activation domains, has three potential myristoylation sites located near the N terminus., and has a characteristic 27 amino acid sequence near the C terminus. And this unique amino acid sequence may cause changes in the ligand-binding affinity and specificity of ERα-36 (74). Studies have reported that neither tamoxifen nor another anti-estrogen, ICI-182,780, failed to block ERα-36-mediated ERK1/2 activation and/or ERα-36 degradation in breast cancer cells. And anti-estrogens may more effectively enhance estrogen signal transduction through ERα-36 to activate ERK1/2. The activation of these pathways contributes to the proliferation of tumor cells (25). Another study reported that tamoxifen was found to bind directly to and activate ERα-36 through transcriptional stimulation of aldehyde dehydrogenase 1A1 (ALDH1A1) that enhanced the stemness and ability to metastasize of breast cancer cells (85). These findings support a potential and profound conflict that depends on the which ER isomer has the predominant expression in HCC tissue, ERα-66 or ERα-36, and may explain why tamoxifen fails to inhibit tumors by antagonizing the estrogen signaling pathway as in breast cancer. Instead, it may bind to ERα-36 and activate the rapid transduction pathway mediated by ERα-36 to promote tumor cell proliferation and metastasis. There is no research report to confirm this inference, and relevant research is needed to confirm it.

So, can the estrogen signaling pathway be used in the treatment of HCC? The answer remains to be determined. Strictly anti-estrogen therapy only antagonizes the estrogen signaling pathway. There are many other treatment strategies that could be based on the estrogen signaling pathway, such as estrogen replacement therapy, a treatment strategy based on promoting the estrogen signaling pathway, or targeting a specific estrogen receptor subtype or other key targets in the estrogen signaling pathway (74). Perhaps one or more of these options might be efficacious in the treatment of HCC.

Menopausal hormone therapy (MHT) is estrogen replacement therapy mainly used to treat menopausal symptoms. In a single-center case-control trial of HCC women and female control subjects, estrogen menopausal hormone therapy reduced HCC and HBV-related HCC, and increased overall survival of the HCC patients (11). This study provides epidemiological evidence for estrogen replacement therapy to prevent the occurrence and development of HCC after menopause.

In a systematic review and dose-response meta-analysis of observational studies on reproductive factors and menopausal hormone therapy related to the risk of primary liver cancer, estrogen exposure was found to be associated with the risk of liver cancer in a J-shaped dose response pattern (12). Late menarche and frequent use of MHT were associated with a reduction in the risk. When women with or without a history of oophorectomy were studied, those that had undergone oophorectomy had an increased risk of liver cancer. This study provides epidemiological support for the protective effect of estrogen in the development of liver cancer in females. At present, there are few comprehensive studies of estrogen replacement therapy in the treatment of liver cancer, most of which are retrospective, and most provide only epidemiological evidence. Therefore, research on the basic mechanism and clinical studies of estrogen replacement therapy in the treatment of liver cancer would be of great value.

In summary, estrogen replacement therapy based on the estrogen signaling pathway for HCC remains a valid and important potential therapeutic option. To more effectively develop anti-liver cancer therapies based on the estrogen signaling pathway, it will be important to explore the phenotypes and molecular subgroups of individuals who benefit most from various interventions.

## 7 Discussion

As an important physiological hormone, estrogen impacts numerous important processes, such as regulating reproductive physiology, cell growth and proliferation, immune response, and metabolism. The estrogen signaling pathway, composed of estrogen and the estrogen receptor, is the main pathway for estrogen to exert its physiological functions. The estrogen signaling pathway is composed of a variety of different estrogen receptor-mediated genomic pathways and rapid transduction pathways. The estrogen receptors and related pathway proteins and effector molecules in the two functional pathways crosstalk with each other. The differences in the transcriptional activity of tissues and physiological processes constitute a complex, multi-functional estrogen signaling pathway.

Epidemiological studies have shown that the incidence and mortality of liver cancer in women are markedly lower than in men, suggesting that the estrogen signaling pathway exerts a protective effect. Recently, evidence has accumulated that has confirmed the anti-liver cancer effect of the estrogen signaling pathway. The estrogen signaling pathway can directly regulate the growth, proliferation, apoptosis and cell cycle of liver cancer cells through genomic and rapid transduction pathways and regulate various cellular stress responses such as cell oxidative stress and autophagy. In addition, tumor microenvironment, non-coding RNA, metabolites and other factors can indirectly regulate tumor growth environment, the immune response to tumors, and epigenetic and genetic changes to exert anti-tumor effects. In addition, the estrogen signaling pathway can directly or indirectly inhibit the progression of hepatitis virus infection and the occurrence and development of hepatitis virus-related liver cancer by regulating host antiviral responses, viral infection pathways, viral replication and transcription.

However, in addition to the inhibitory effect of the estrogen signaling pathway on liver cancer, some studies have also reported promotion of liver cancer by the estrogen signaling pathway. Analysis of these controversial results has revealed that the promotion of estrogen on liver cancer may be mediated by the new estrogen receptor ERα-36 and estrogen-related receptor γ (ERRγ). Analysis of the estrogen signaling pathway and the functional pathway mediated by the estrogen receptor requires analysis of the composition of a specific molecular subgroup of a specific estrogen receptor in a specific environment.

Anti-estrogen therapy based on the anti-estrogen signaling pathway (such as tamoxifen) applied to the treatment of liver cancer has not previously achieved satisfactory results. This may be attributed to the main molecular target being ERα-66, while HCC typically has low expression of ERα-66. Moreover, ERα-66 in HCC usually mediates anti-liver cancer effects. Epidemiological studies have now provided strong basis for the protective effect of estrogen replacement therapy/estrogen therapy based on the estrogen signaling pathway therapy in female liver cancer patients.

In the future, we plan to specifically explore the phenotypes and molecular subgroups of individuals who benefit most from various interventions (such as estrogen therapy). This should facilitate selection of specific interventions for individual patients to obtain maximum benefit. This anti-liver cancer treatment should be directed toward personalized, precision treatment.

Based on previously published reports, we have summarized, analyzed, explored and predicted the potential targets and mechanisms of the estrogen signaling pathway for anti-liver cancer therapy in the future. This should provide a strong theoretical basis for subsequent in-depth research and clinical application. The most appealing initial candidate would be estrogen therapy based on the treatment strategy of the estrogen signaling pathway. Although it has been epidemiologically characterized as having a protective effect in liver cancer, a large randomized multicenter controlled trial would be needed to appropriately answer the efficacy of estrogen therapy on liver cancer. Equally important, because the estrogen signaling pathway is composed of different estrogen receptors and complex functional pathways, it will be necessary to explore which estrogen receptor phenotype and molecular subgroups of liver cancer patients can obtain the most optimal outcomes from estrogen therapy. Gender differences, menopausal status and obesity related to estrogen therapy also deserve further evaluation.

A second treatment strategy could be based on specific estrogen receptor subtypes in the estrogen signaling pathway. Three estrogen receptors, ERα-66, ERβ and GPER, are principally responsible for mediating the anti-tumor effects in liver cancer. In the future, specific agonists developed for these targets have great potential for the clinical treatment of liver cancer. Because ERα-36 has a tumor-promoting effect in liver cancer, and it is highly expressed in cirrhotic and liver cancer tissues, it has great potential as a biomarker for liver cancer diagnosis, and antagonists targeting ERα-36 are expected to become a new type of therapy for liver cancer. The above specific target treatments need to be based on the development or application of more accurate, reliable and convenient estrogen receptor isoform detection methods. Monitoring of changes in estrogen receptor subtypes after treatment will facilitate the precision, efficacy analysis and prognostic evaluation of targeted therapy.

Finally, treatment strategies based on specific key molecules/inflammatory mediators/regulatory pathways in the estrogen signaling pathway also have great potential. The estrogen signaling pathway can regulate the body’s inflammatory response to inhibit the progression of liver cancer. The key inflammatory mediators such as IL-6, IL-1α, and IL-1β are expected to become therapeutic targets. The estrogen signaling pathway can regulate the tumor microenvironment of liver cancer through a variety of ways. In the future, can we use the special immune regulation ability of the estrogen signaling pathway to combine with existing tumor immunotherapy to enhance efficacy and overcome problems of acquired drug resistance. This will require a comprehensive and detailed clarification of the regulation mechanism of the estrogen signaling pathway on the tumor microenvironment. In this way, we can distinguish whether the estrogen signaling pathway regulated by different estrogen receptors and functional pathways activates the tumor-promoting microenvironment or the tumor suppressing microenvironment. More precise regulation of tumor microenvironment, combined with tumor immunotherapy should offer a comprehensive plan to obtain maximum benefit.

## Author Contributions

YG, GW, and JY wrote and edited this manuscript and created figures. LM and XC reviewed and revised the manuscript. LM and XY provided direction and guidance throughout the preparation of the manuscript. QY, WJ, and SL participated in the review and revision of the manuscript during the review. All authors contributed to the article and approved the submitted version.

## Funding

The authors appreciate the financial support from Natural Science Foundation of China (81800774), Pearl River S&T Nova Program of Guangzhou (201806010166), Natural Science Foundation of Guangdong Province (2214050007824), China Postdoctoral Science Foundation (2017M622660).

## Conflict of Interest

The authors declare that the research was conducted in the absence of any commercial or financial relationships that could be construed as a potential conflict of interest.

## Publisher’s Note

All claims expressed in this article are solely those of the authors and do not necessarily represent those of their affiliated organizations, or those of the publisher, the editors and the reviewers. Any product that may be evaluated in this article, or claim that may be made by its manufacturer, is not guaranteed or endorsed by the publisher.
